# The Optimal Carbon Emission Reduction and Prices with Cap and Trade Mechanism and Competition

**DOI:** 10.3390/ijerph15112570

**Published:** 2018-11-16

**Authors:** Linghong Zhang, Hao Zhou, Yanyan Liu, Rui Lu

**Affiliations:** 1 Management Science and Engineering Postdoctoral Mobile Station, Shandong Normal University, Ji’nan 250014, China; lhzhang@sdnu.edu.cn; 2 Antai College of Economics and Management, Shanghai Jiaotong University, Shanghai 200030, China; 3 Business School, Shandong Normal University, Ji’nan 250014, China; 2017020943@stu.sdnu.edu.cn (H.Z.); liu2313343358@126.com (Y.L.); 4 School of Economics and Management, Hangzhou Normal University, Hangzhou 311121, China

**Keywords:** Carbon cap and trade mechanism, carbon emission reduction, manufacturer size, consumer environment awareness, competition

## Abstract

More and more countries employ the Carbon Cap and Trade mechanism (CCT-mechanism) to stimulate the manufacturer to produce much more eco-friendly products. In this paper, we study how the CCT-mechanism affects competitive manufacturers’ product design and pricing strategies. Assume that there are two competitive manufacturers; we give the optimal closed form solutions of the carbon emission reduction rates and retail prices in the Nash game model and the Stackelberg game model with CCT-mechanism, respectively. Additionally, we also discuss the impacts of CCT-mechanism, consumer environmental awareness (CEA), and the sensitivity of switchovers toward price on the optimal carbon emission reduction rates, retail prices, and manufacturers’ profits. We find that (i) when the carbon quota is not enough, there is a trade off between investing in producing much greener product and purchasing carbon quota; when the carbon price is not high, the manufacturer tends to purchase the carbon quota; and when the carbon price is much higher, the manufacturer is more willing to increase the environmental quality of the product; (ii) manufacturer’s size affects product’s emission reduction rate and manufacturer’s optimal profit; larger manufacturer tends to produce much greener product, but it does not mean that he could obtain much more money than the small manufacturer; and (iii) the decision sequence changes manufacturer’s strategies; the optimal emission reduction rate in Nash and Stackelberg game models are almost the same, but the differences of prices and profits between Nash and Stackelberg model’s are much bigger.

## 1. Introduction

Greenhouse Gases (GHGs) is the main factor that induces climate change. Many countries have attempted to design market-based carbon trading mechanism to control carbon emission. Carbon trading mechanism pose new challenges to enterprises and make enterprise operations management more complex. For example, Ahmed and Sarker [1] presented that firms need to reallocate for agricultural zones and bio-refineries to decrease the carbon emission of the biofuel supply chain. Meneghetti and Monti [2] showed that the yearly rate of refrigeration energy is regularly increasing, which in turn increases the carbon footprint, an optimization model for the sustainable design of refrigerated automated storage and retrieval systems need to be proposed. Therefore, with Carbon Cap and Trade mechanism and manufacturers’ competition, how manufacturers adjust their strategies, especially the product design and pricing strategies, becomes an urgent problem.

In the past decades, Carbon Cap and Trade mechanism have been launched and developed (Kyoto Protocol, 1997; The Copenhagen Accord, 2009; The Doha Amendment, 2012) [3,4,5]. In the Carbon Cap and Trade mechanism, firms can get the total amount of carbon quotas (carbon cap) from the government and can sell/purchase carbon quotas in carbon emission trading market when they need less/more carbon quotas. Carbon emission trading implies profound energy and economic effects with significant policy implications (see for instance, Tol et al., Edenhofer et al., Knopf et al., Hübler et al., Capros et al., and Riahi et al. [6,7,8,9,10,11]). Some firms have begun to invest in technologies to reduce carbon emissions. For example, Walmart has adopted many new technologies to curb carbon emissions, including designing and opening energy efficient stores (Plambeck, 2012) [12]. While carbon trading is increasingly prevalent in the international market, whether and how CCT-mechanism affects manufacturers’ decisions still needs to investigate and research on this field still be very scare.

In addition to the CCT-mechanism, consumer environmental awareness plays an important role in motivating manufacturers to reduce carbon emissions. According to a survey from BBMG Conscious Consumer Report, 67% of Americans think that it is very important to purchase products that benefit the environment, and 51% of them are willing to pay higher prices for environmentally-friendly products [13]. Manufacturers may increase the green level of products by increasing investment in emission reductions to attract environmentally-friendly consumers. Therefore, the impact of CEA on manufacturers’ decision-making is another focus of our research.

The aim of this paper is to study how the CCT-mechanism and CEA affect two competitive manufacturers’ product design and price strategies. Therefore, we mainly address the following questions:

(i) How the CCT-mechanism, including the carbon quota and carbon price, affect competitive manufacturers’ strategies, such as the carbon emission reduction rates and retail prices?
(ii) How manufacturers’ action sequences (Nash game and Stackelberg game) affect manufacturers’ strategies?
(iii) Will CEA and consumer’s sensitivity of switchovers toward price change manufacturer’s strategies?

To solve these questions, we assume that there are two manufacturers: Manufacturer 1 and manufacturer 2. Each manufacturer produces one product with environmental quality (low carbon emission). Additionally, manufacturer *i* (*i* = 1, 2) produces product *i*. Consumer demand is affected by the carbon emission reduction rate, price, and CEA. Each manufacturer’s carbon emission allocation cap is determined by both manufacturers’ sizes. We discuss two game models: (1) The Nash game model where two manufacturers determine a products’ carbon emission reduction rates and their prices simultaneously; and (2) the Stackelberg game model where manufacturer 1 as the leader acts first to determine the optimal carbon emission reduction rate and price of product 1, then manufacturer 2 follows to determine the carbon emission reduction rate and price of product 2. We give the optimal closed form solutions of the carbon emission reduction rates and retail prices by using the backward induction method in the Nash game model and Stackelberg game model, respectively. Additionally, we study the impact of CCT-mechanism, carbon price, CEA, and the sensitivity of switchovers toward the price on the optimal carbon emission reduction rates, retail prices, and manufcturers’ profits. Finally, we perform the comparisons between two models and present the sensitivity analyses on model parameters with numerical examples.

This study extends prior literature in three important aspects. First, we consider both the product design and price strategies simultaneously, while some literature that focuses on the CCT-mechanism and competition only study the optimal price (Zhang and Xu, Xu et al., and Chen et al. [3,14,15]). Second, we study how competition affects low-carbon manufacturers’ strategies, but most of them focus on one manufacturer (Xu et al., Ji et al., and Xu et al. [16,17,18]). Third, we study the impact of manufacturers’ decision sequences on manufacturers’ strategies by considering both the Nash game and the Stackelberg game, while most of other literature only consider the Stackelberg game (Ji et al., Hammami et al., and Zhu et al. [17,19,20]).

The remainder of the paper is organized as follows. We review the existing literature related to environmental policy, the influence of CEA on supply chain enterprises and the influence of competition on supply chain enterprises. Section 3 provides the assumptions and the optimization model for manufacturers decision-making under CCT-mechanism. Section 4 provides model formulations and solutions, then gives some propositions. Section 5 presents the sensitivity analyses on model parameters with numerical examples. Section 6 summarizes our main findings and gives some managerial insights. All proofs are relegated to the Appendix A.

## 2. Literature Review

There is some literature related to green product, we here primarily review the most related three research streams: CCT-mechanism, CEA, and manufacturer competition.

The first stream focuses on how CCT-mechanism affects the manufacturer’s strategies. Dong et al. [21] studied the sustainability investment on sustainable product consideration for decentralized and centralized supply chains under CCT-mechanism, they derived the optimal order quantity and sustainability investment, and found the sustainability investment efficiency has a significant impact on the optimal solutions. Bird et al. [22] summarized key issues for renewable energy markets that are emerging with carbon regulation and explores policy options under consideration for designing carbon policies to enable carbon markets and renewable energy markets to work together. Gong and Zhou [23] presented the optimal production strategy under carbon trading policy through a dynamic model from the view of pricing and production decisions. Chaabane et al. [24] designed the closed supply chain under carbon trading regulation and analyzed the cap-and-trade policy. Chang et al. [25] studied how the CCT-mechanism affects the monopoly manufacturer’s the production decisions in a hybrid manufacturing-remanufacturing system. Gracia et al. [26] issued the relationship between cap and trade regulation and inventory, and found that carbon prices has a great effect on decisions. Dou [27] indicated that low carbon economy development needs the corresponding conditions such as carbon finance and low carbon policies by building a cone model. Cheng et al. [28] showed that high-strength carbon policies don’t necessarily encourage enterprises to effectively reduce carbon emissions and the policy sensitivity of the carbon trading mechanism is more obvious than the carbon tax. Yang et al. [29] studied the issue of how to allocate the carbon emissions quotas among different industrial sectors fairly and effectively. Jiang et al. [30] explored the initial allocation of carbon emission permits among the provinces of China from the perspective of fairness and constructed a model of the initial inter-provincial allocation of carbon emission permits. Chen et al. [15] investigated the manufacturing, remanufacturing, and collection decisions of a monopoly manufacturer under cap-and-trade regulation and take-back regulation conditions. However, above literature did not consider the impact of CEA and most of the studies did not explore the manufacturer’s competition.

The second stream focuses on the impact of CEA on supply chain enterprises. Liu et al. [31] focused on the impacts of competition and CEA on key supply chain players by the use of two-stage Stackelberg game models and found that as CEA increases, retailers and manufacturers with superior eco-friendly operations will be benefited. Zhang, Wang, and You [32] investigated the impact of CEA on order quantities and channel coordination within a one-manufacturer and one-retailer supply chain. They concluded that the retailer’s profit monotonically increases, while the manufacturer’s profit is convex with respect to CEA and order quantity of the environmental product increases with CEA. Xu and Xie [33] constructed a two-stage closed-loop supply chain and their study shows that under both decentralized and centralized decision-making models, the products’ eco-friendly level, return rate, and profit of the node enterprise are positively correlated to the CEA. Hammami, Nouira, and Frein [19] studied the effect of CEA on the emission intensity and price of a product. Their main results indicated that CEA is an efficient driver for better environmental performance. However, the above literature did not consider the impact of environment policies.

The third stream focuses on the impact of competition on supply chain enterprises. For instance, Moorthy, Banker et al., Hall and Porteus, Tsay, and Agrawal [34,35,36,37], etc. These studies found that, as competition intensifies, the equilibrium levels of quality and service increase, while price and delivery time decrease. Chen, Luo, and Wang [38] considered price and carbon emission in establishing the demand model, and incorporated competition between the two rival manufacturers in the demand function. However, the above literature did not consider the impacts of CCT-mechanism.

More papers are about sustainable supply chain management, readers could further read Seuring and Muller and Shashi et al. [4,5], which offers a conceptual framework to summarize the research in this field.

In sum, most studies did not consider CCT-mechanism, CEA and manufacturers competition simultaneously, hence in this paper we will not only incorporate CCT-mechanism, but also consider the impact of competition and CEA on supply chain.

We summarize the papers that are most related to our research in Table 1.

## 3. Problem Assumptions and Model Description

Assume that there are two environmental manufacturers: Manufacturer 1 (denote as $m_{1}$) and manufacturer 2 (denote as $m_{2}$). Manufacturer *i* produces product *i* and the two products are substitutable (*i =* 1, 2). Then, we suppose that each product has two attributes influencing consumer demand: Emission reduction rate (denoted as e) and price (denoted as p) [32]. Product demand increases with emission reduction rate and decreases with price.

**Figure 1:**
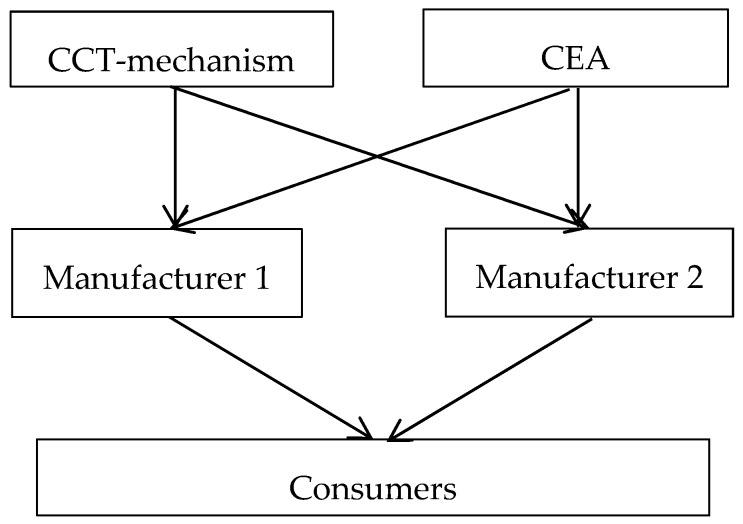
The supply chain structure and decision process.

Assume that the government’s carbon cap is constant and the government allocates a carbon cap to each manufacturer according to their sizes. The larger manufacturer’s size, the larger carbon cap. The manufacturer can buy or sell carbon credit on a trading market of carbon emission to meet its carbon emission needs and to maximize his profit. When the manufacturer chooses to produce low carbon emission product and his carbon credit has surplus, then he can sell the remaining carbon quota to another manufacturer to obtain the extra revenue. However, if the manufacturer produces a product with a high carbon emission, he may need purchase carbon quota for the excess carbon emission. With the CCT-mechanism, the manufacturers needs to determine the optimal carbon emission reduction rates and prices of the products. Figure 1 shows basic supply chain structure and decision process of this paper.

Similar to Zhang et al. [32,39,40], we consider that the demand functions for product *i* (*i =* 1, 2) denoted by $q_{i}$, therefore, the demand functions of manufacturer *i* is as follows:

(1) $$q_{1}=a-p_{1}+\theta p_{2}-\tau \theta e_{2}+\tau e_{1}\;$$

(2) $$q_{2}=a-p_{2}+\theta p_{1}-\tau \theta e_{1}+\tau e_{2}\;$$

where *a* is initial market potential, *τ* represents CEA, θ(0≤θ≤1) represents the sensitivity of switchovers toward price. Table 2 summarizes the major notations we will use in our model development.

A higher amount of emission reduction raises the difficulty of reducing carbon emissions such that a higher cost is incurred [17]. Similar to Ji et al., Zhu and He, and Wang et al. [17,20,41], we model the cost for the carbon emission reduction rate *e* as an increasing quadratic function as $ce^{2}$, and the constant *c* is the cost coefficient, independent of the production volume, and is a strictly positive cost coefficient.

Due to the government’s carbon quota being distributed according to the manufacturer’s size, manufacturer *i* has the carbon quota $\frac{M_{i}}{M_{1}+M_{2}}s_{0}$, where $M_{i}$ represents manufacturer *i*’s size, and $s_{0}$ is the total carbon cap. The total amount of the carbon emission of manufacturer *i* is $M_{i}\left(1-e_{i}\right)$. According to the above assumptions and demand functions, the profit function of manufacturer *i* denoted by $\pi_{i}$, can be modeled as follows:

(3) $$\pi_{1}=p_{1}\left(a-p_{1}+\theta p_{2}+\tau e_{1}-\tau \theta e_{2}\right)-ce_{1}^{2}+\left(\frac{M_{1}}{M_{1}+M_{2}}s_{0}-M_{1}\left(1-e_{1}\right)\right)c_{0}\;$$

(4) $$\pi_{2}=p_{2}\left(a-p_{2}+\theta p_{1}-\tau \theta e_{1}+\tau e_{2}\right)-ce_{2}^{2}+\left(\frac{M_{2}}{M_{1}+M_{2}}s_{0}-M_{2}\left(1-e_{2}\right)\right)c_{0}\;$$

## 4. Model Formulation and Solution

In this section, we explore the impacts of different game models on manufacturers’ optimal carbon emission reduction rates and prices. First, we present the Nash equilibrium model (denoted by N), and then we study the Stackelberg game model (denoted by S).

### 4.1. Nash Equilibrium Model

In this section, we assume two manufacturers take actions simultaneously: In the first period, the two manufacturers determine the carbon emission reduction rates simultaneously; in the second period, they determine the retail prices at the same time. We use the backward induction method to solve the Nash equilibrium model. The N on the upper right represents the Nash equilibrium model.

Similar to Xu et al., Dong et al., and Yi et al. [14,21,42], we put forward the following assumption.

**Assumption 1.**  *Cost coefficient of emission reduction of the manufacturer and the consumer environmental awareness satisfy*$c>\tau^{2}/4$.

In practice, the R&D cost is very high, so we assume that c is larger than a threshold value. Of course, we can also obtain analytical results even if without this assumption. Specifically, if $c\leq \tau^{2}/4$, the optimal solutions are the lower bound or upper bound of the emission reduction rates of the manufacturer, then the manufacture will not invest on the green level of production or a very high green level. In order to avoid these trivial cases and make our results more elegant, we present assumption 1 to guarantee that profit functions are concavity.

#### 4.1.1. The Optimal Retail Prices

In this section, we present the optimal retail price and discuss the impact of cost coefficient of emission reduction, the government’s total carbon quota, carbon price, and the size of manufacturers on the optimal prices and profits of the two manufacturers.

**Theorem 1.**  *The optimal retail price are determined by*

(5) $$p_{1}^{N*}=\frac{\left(2+\theta \right)a+e_{1}\tau (2-\theta^{2})-e_{2}\tau \theta }{4-\theta^{2}}\;$$

(6) $$p_{2}^{N*}=\frac{\left(2+\theta \right)a+e_{1}\tau (2-\theta^{2})-e_{2}\tau \theta }{4-\theta^{2}}\;$$

For each manufacturer, the optimal retail price $p_{1}^{N*}$ and $p_{2}^{N*}$ are global optimal solution, according to the results of Theorem 1, we can get some Propositions.

**Proposition 1.** 

*(i)*  *the manufacturer 1’s optimal retail price*$p_{1}^{N*}$increases with$e_{1}$*and decreases with*$e_{2}$;
*(ii)*  *the manufacturer 2’s optimal retail price*$p_{2}^{N*}$increases with$e_{2}$*and decreases with*$e_{1}$.

Proposition 1 means that the manufacturer’s optimal retail price increases with its own emission reduction rate and decreases with the emission reduction rate of the rival manufacturer.

**Proposition 2.** 

*(i)*  *if*$\frac{e_{1}}{e_{2}}\geq \frac{\theta }{2-\theta^{2}}$*, the manufacturer 1’s optimal retail price*$p_{1}^{N*}$increases withτ*; the manufacturer 2’s optimal retail price*$p_{2}^{N*}$decreases withτ.
*(ii)*  *if*$\frac{e_{1}}{e_{2}}<\frac{\theta }{2-\theta^{2}}$*, the manufacturer 1’s optimal retail price*$p_{1}^{N*}$decreases withτ*; the manufacturer 2’s optimal retail price*$p_{2}^{N*}$increases withτ.

Proposition 2 presents when the ratio between emission reduction rates of manufacturer 1 and manufacturer 2 is higher than $\frac{\theta }{2-\theta^{2}}$, then the manufacturer 1’s optimal retail price increases with CEA; if the ratio between emission reduction rates of manufacturer 1 and that of manufacturer 2 is lower than $\frac{\theta }{2-\theta^{2}}$, the manufacturer 1’s optimal retail price decreases with CEA. Similarly, when the ratio between emission reduction rates of manufacturer 2 and that of manufacturer 1 is higher than $\frac{\theta }{2-\theta^{2}}$, the manufacturer 2’s optimal retail price increases with CEA; if the ratio between emission reduction rates of manufacturer 2 and that of manufacturer 1 is lower than $\frac{\theta }{2-\theta^{2}}$, the manufacturer 2’s optimal retail price decreases with CEA because each product has environmental quality, and each product’s demand will change with CEA. In other word, when CEA is much larger, the consumer may purchase the product with high environmental quality, thus the demand and the price of the product with low environmental quality will decrease. The price change depends on the difference of the environmental quality between two products and the sensitivity of switchovers toward price.

**Proposition 3.**  *The optimal profits of manufacturer 1 and manufacturer 2 increases with*$s_{0}$.

Proposition 3 means that each manufacturer’s profit increases with the total carbon quota.

**Proposition 4.** 

*(i)*  *if*$1-\frac{s_{0}}{M_{1}+M_{2}}\leq e_{i}\leq 1$*, then the optimal profit of manufacturer i increases with*$c_{0}$;
*(ii)*  *if*$0\leq e_{i}<1-\frac{s_{0}}{M_{1}+M_{2}}$*, then the optimal profit of manufacturer i decreases with*$c_{0}$;

Proposition 4 presents emission reduction rates of manufacturer i, $e_{i}\in \left[1-\frac{s_{0}}{M_{1}+M_{2}},1\right]$, the optimal profit of manufacturer i increases with unit carbon price; if the emission reduction rates of manufacturer i, $e_{i}\in \left[0,1-\frac{s_{0}}{M_{1}+M_{2}}\right)$, then, the optimal profit of manufacturer i decreases with unit carbon price (i=1, 2). In other word, if the carbon emission rate is much higher, then the manufacturer needs to purchase the carbon quota, thus the manufacturer’s profit decreases with carbon price; otherwise, if the carbon emission rate is much lower, then the manufacturer’s profit increases with carbon price.

**Proposition 5.** 

*(i)*  *the optimal profit of manufacturer 1 decreases with*$M_{2}$;
*(ii)*  *the optimal profit of manufacturer 2 decreases with*$M_{1}$.

Proposition 5 means that the optimal profit of manufacturer i decreases with the size of the rival manufacturer. As the carbon quota is allocated according to the manufacturer’s size, then large manufacturer’s size means high carbon quota, hence the manufacturer’s profit decreases with rival manufacturer’s size.

#### 4.1.2. The Optimal Emission Reduction Rates

In Section 4.1.1, we give the optimal retail price of manufacturer $p_{1}^{N*}$ and $p_{2}^{N*}$, the optimal profit $\pi_{1}^{N*}$ and $\pi_{2}^{N*}$. In this section, we will give the optimal carbon emission reduction rates based on the optimal retail prices and profits’ functions.

**Theorem 2.**  *According to Theorem 1 and Theorem 2, the optimal carbon emission reduction rate are determined by*

(7) $$e_{1}^{N*}=\frac{\begin{array}{l} 2a\tau^{3}\left(\theta^{5}+\theta^{4}-4\theta^{3}-4\theta^{2}+4\theta +4\right)+c_{0}\tau^{2}\left(-M_{1}\theta^{6}+M_{2}\theta^{5}+8M_{1}\theta^{4}-6M_{2}\theta^{3}-20M_{1}\theta^{2}+8M_{2}\theta +16M_{1}\right)\\ +2ac\tau \left(-\theta^{5}-2\theta^{4}+6\theta^{3}+12\theta^{2}-8\theta -16\right)+M_{1}cc_{0}\left(\theta^{6}-12\theta^{4}+48\theta^{2}-64\right) \end{array}}{2\left[c^{2}\left(\theta^{6}-12\theta^{4}+48\theta^{2}-64\right)+2c\tau^{2}\left(-\theta^{6}+8\theta^{4}-20\theta^{2}+16\right)+\tau^{4}\left(\theta^{6}-5\theta^{4}+8\theta^{2}-4\right)\right]}\;$$

(8) $$e_{2}^{N*}=\frac{\begin{array}{l} 2a\tau^{3}\left(\theta^{5}+\theta^{4}-4\theta^{3}-4\theta^{2}+4\theta +4\right)+c_{0}\tau^{2}\left(-M_{2}\theta^{6}+M_{1}\theta^{5}+8M_{2}\theta^{4}-6M_{1}\theta^{3}-20M_{2}\theta^{2}+8M_{1}\theta +16M_{2}\right)\\ +2ac\tau \left(-\theta^{5}-2\theta^{4}+6\theta^{3}+12\theta^{2}-8\theta -16\right)+M_{2}cc_{0}\left(\theta^{6}-12\theta^{4}+48\theta^{2}-64\right) \end{array}}{2\left[c^{2}\left(\theta^{6}-12\theta^{4}+48\theta^{2}-64\right)+2c\tau^{2}\left(-\theta^{6}+8\theta^{4}-20\theta^{2}+16\right)+\tau^{4}\left(\theta^{6}-5\theta^{4}+8\theta^{2}-4\right)\right]}\;$$

Through our calculation process, the optimal carbon emission reduction rates $e_{1}^{N*}$ and $e_{2}^{N*}$ are the global optimal solution, because of the complexity of the Equations (7) and (8), we will study the effect of CEA, the sensitivity of switchovers toward price θ and manufacturer’s size M on the optimal emission reduction rate in Section 5.

### 4.2. Stackelberg Game Model

In this section, we assume Manufacturer 1 is the leader and manufacturer 2 is the follower. We use backward induction method to determine the optimal solutions. Firstly, manufacturer 2 determines its carbon emission reduction rate and retail price; secondly, manufacturer 1 chooses its carbon emission reduction rate and retail price to maximize his profit. The S on the upper right denotes the Stackelberg game model.

Similar to Ji et al. and Wang et al. [17,41], in order to guarantee the profit functions’ concavity, we present the following assumption.

**Assumption 2.**  *Consumer’s environmental awareness, cost coefficient of emission reduction and the sensitivity of switchovers toward price satisfies ${\left(4c-\tau^{2}\right)}^{2}+\tau^{2}\theta^{2}(2c-\tau^{2})>0$.*

According to Assumption 2, we can obtain the following theorems and propositions.

#### 4.2.1. The Optimal Carbon Emission Reduction Rate and Retail Price of Manufacturer 2

In this section, we present manufacturer 2’s optimal carbon emission reduction rate and retail price, then discuss the change trends with some parameters.

**Theorem 3.**  *The optimal carbon emission reduction rate and retail price of manufacturer 2 in the Stackelberg game model are as follows*

(9) $$e_{2}^{S*}=\frac{2M_{2}c_{0}+a\tau -e_{1}\tau^{2}\theta +p_{1}\tau \theta }{4c-\tau^{2}}\;$$

(10) $$p_{2}^{S*}=\frac{2ac+M_{2}c_{0}\tau +2cp_{1}\theta -2ce_{1}\tau \theta }{4c-\tau^{2}}\;$$

Similarly, Section 4.1, in this section, shows the optimal emission reduction rate and retail price of manufacturer 1 and manufacturer 2 are the global optimal solutions. According to the results of Theorem 4, we can get Propositions 6–9.

**Proposition 6.**  *The optimal carbon emission reduction rate and retail price of manufacturer 2 increase with*$c_{0}$.

When the carbon price increases, the green quality of the product increases no matter whether there is a sufficient carbon cap. As the total carbon quota is fixed, the manufacturer may actively reduce his carbon emission and sell the remaining carbon quota to other manufacturer to obtain extra revenue.

**Proposition 7.**  *The optimal carbon emission reduction rate and retail price of manufacturer 2 increase with*$M_{2}$.

Proposition 7 means that the larger the size of manufacturer, the greener product the manufacturer produces. In other word, if the manufacturer has a big size, it tends to invest more to improve the carbon emission reduction rate to win more consumers, and the retail price can be appropriately increased.

**Proposition 8.**  *The optimal carbon emission reduction rate and retail price of manufacturer 2 decrease with *$e_{1}$*and increase with*$p_{1}$.

If manufacturer 1 as a dominant player in the game chooses to increase its emission reduction rate, manufacturer 2 as a follower in the game will purchase carbon quota instead of increasing the investment of carbon emission reduction, therefore manufacturer 2 could lower its retail price appropriately to gain competitive advantage. If manufacturer 1 raises its retail price, manufacturer 2 as a follower also could raise the retail price to obtain more profits.

**Proposition 9.** 

*(i)*  *when*$\frac{p_{1}}{e_{1}}<2\sqrt{c}$, *and*
  *(a)*  *if*$\tau \leq \frac{p_{1}}{e_{1}}$, *then the optimal carbon emission reduction rate and the optimal retail price of manufacturer 2 increase with*θ;
  *(b)*  *if*$\frac{p_{1}}{e_{1}}<\tau <2\sqrt{c}$, *then the optimal carbon emission reduction rate and the optimal retail price of manufacturer 2 decrease with*θ;
*(ii)*  *when*$\frac{p_{1}}{e_{1}}\geq 2\sqrt{c}$, *the optimal carbon emission reduction rate and the optimal retail price of manufacturer 2 increase with*θ.

Proposition 9 presents the change trends of the optimal carbon emission reduction rate and the optimal retail price of manufacturer 2 with ratio of the retail price to the emission reduction rate of product 1 and CEA. Manufacturer 2 as a follower, if the ratio of product 1 and CEA are not very high, then manufacturer 2’s optimal carbon emission reduction rate and the optimal retail price increase with θ; if the ratio of product 1 is not very high and CEA is not very low, then manufacturer 2’s optimal carbon emission reduction rate and the optimal retail price increase with θ. When the ratio of product 1 is not very low, then manufacturer 2’s optimal carbon emission reduction rate and the optimal retail price increase with θ.

#### 4.2.2. The Optimal Retail Price and Emission Reduction Rate of Manufacturer 1

In this section, we derive the optimal retail price and emission reduction rate of manufacturer 1 based on the optimal retail price and emission reduction rate of manufacturer 2.

**Theorem 4.**  *The optimal retail prices of manufacturer 1 in the Stackelberg game model are as follows:*

(11) $$e_{1}^{S*}=\frac{8M_{1}cc_{0}+4ac\tau +2ac\tau \theta -a\tau^{3}-a\tau^{3}\theta -2M_{1}c_{0}\tau^{2}-M_{2}c_{0}\tau^{2}\theta }{16c^{2}+2c\tau^{2}\theta^{2}-8c\tau^{2}-\tau^{4}\theta^{2}+\tau^{4}}\;$$

(12) $$p_{1}^{S*}=\frac{\left(\tau^{2}-4c\right)\left(-4ac^{2}\theta -8ac^{2}+2ac\tau^{2}\theta +2ac\tau^{2}+2M_{1}c_{0}c\tau \theta^{2}+2M_{2}c_{0}c\tau \theta -4M_{1}c_{0}c\tau -M_{1}c_{0}\tau^{3}\theta^{2}+M_{1}c_{0}\tau^{3}\right)}{\left(\tau^{2}\theta^{2}-\tau^{2}-2c\theta^{2}+4c\right)\left(16c^{2}+2c\tau^{2}\theta^{2}-8c\tau^{2}-\tau^{4}\theta^{2}+\tau^{4}\right)}\;$$

Same as Theorem 2, because of the complexity of the Equations (11) and (12), we will study the impacts of CEA, the sensitivity of switchovers toward price and the size of manufacturer on the optimal emission reduction rates, retail prices and profits of manufacturer i(i=1, 2) in the numerical examples.

## 5. Numerical Examples

To draw more managerial insights from the theoretical results above, we present the numerical analysis in this section. We mainly compare the optimal solutions between Nash game model and Stackelberg model, and discuss the effect of carbon price, CEA, manufacturer’s size, and the sensitivity of switchovers toward price on optimal emission reduction rates, the optimal retail prices and manufacturers’ profits. Due to the difficulty of acquiring accurate data from the manufacturers, we set some estimated parameters to present the influence of several parameters on the optimal solutions and profits. Similar to Zhang and Xu, Ji et al., Xu et al., and Yang et al. [3,17,18,43], let a=10, c=50, $s_{0}=4$. The results obtained by Nash equilibrium model are denoted by N, and that of Stackelberg game model denoted by S, manufacturer 1 denoted by 1, manufacturer 2 denoted by 2.

### 5.1. Impacts of Carbon Price on Optimal Emission Reduction Rates, Retail Prices and Profits

**Figure 2:**
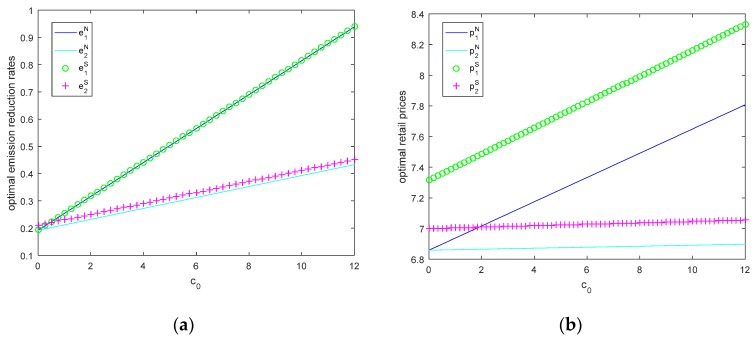
(**a**) Optimal emission reduction rates and (**b**) optimal retail prices as a function of c0 when the total carbon quota is insufficient and sufficient.

**Figure 3:**
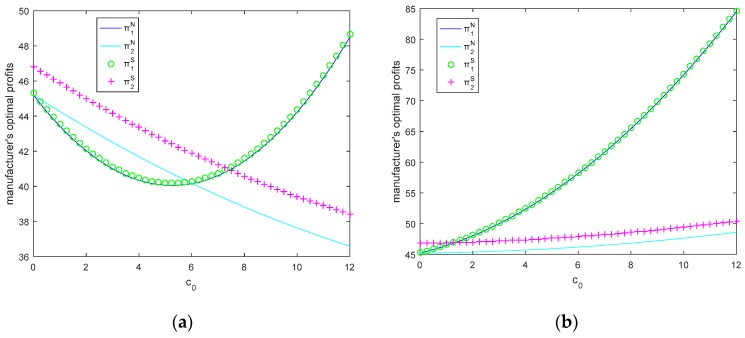
Manufacturer’s optimal profits as a function of c_0_ when total carbon quota is (**a**) insufficient and (**b**) sufficient.

In this section, let θ=0.5, τ=3, $M_{1}=6$, $M_{2}=2$, we will study the changes of optimal emission reduction rates, the optimal retail prices, and optimal profits with carbon price when the government’s total carbon quota is insufficient and sufficient in Figure 2 and Figure 3.

According to Equations (5)–(12) and Figure 2, we can see that manufacturer’s optimal emission reduction rates and retail prices increase with carbon price $c_{0}$ and does not change with the total carbon quota in both models. Due to when the carbon price increases, manufacturers will purchase the carbon right with high cost or sell the carbon right with high revenue, hence high carbon price makes manufacturers produce much greener products.

When $c_{0}=0$, it represents that there is no CCT-mechanism. From Figure 2. We can see that the optimal emission reduction rates and the retail prices without CCT-mechanism are lower than those with CCT-mechanism. In other words, CCT-mechanism could stimulate the firms to provide the product with low emission, and as carbon price increases, firms are willing to produce much lower emission products.

While the optimal carbon emission reduction rates of manufacturer *i* are almost the same in the Nash model and in Stackelberg model, and the prices vary widely in the two models. Therefore, the different decision sequence affects prices significantly. Additionally, the larger size of the manufacturer, the greater change of the emission reduction rate and retail price.

From Figure 3b, we can see that two competitive manufacturers’ optimal profits increase with *c*_0_ in both models when the government’s total carbon quota is sufficient. The higher price, the more profit, because the manufacturer could sell the remaining carbon quota to other manufacturers to obtain the extra revenue. From Figure 3a, it is interesting to see that as carbon price increases, manufacturer 1’s optimal profit first decreases, then increases, but manufacturer 2’s profit decreases all the time. As carbon price increases, at first manufacturer 1 as a large manufacturer could invest less in increasing the environmental quality of product 1 and pay for the increasing of the carbon price when the carbon quota is not sufficient, however, as carbon price increases, the large manufacturer tends to invest much more in increasing the environmental quality of product 1 than purchasing the carbon quota. In other words, there is a trade off between investing in green product and purchasing carbon quota. When the carbon price is not high, the manufacturer tends to purchase the carbon quota, and when the carbon price is much higher, the manufacturer is more willing to increase the environmental quality of the product. For manufacturer 2 (a smaller manufacturer), although increasing the environmental quality of the product with carbon price, the total carbon quota of manufacturer 2 is still not enough because of the size limitation, and hence, manufacturer 2’s profit decreases with carbon price.

We also find that when the carbon price is not very high (*c*_0_ < 6 in Nash model, *c*_0_ < 7.3 in Stackelberg model), the smaller manufacturer obtains more profit than that of the larger manufacturer; only when the carbon price is very high, the larger manufacturer will obtain more profit than that of the smaller manufacturer. In other words, a small manufacturer does not mean that he obtains less profit. Another interesting finding is Stackelberg game is more beneficial to small manufacturer compared with the Nash game.

Therefore, when the carbon price is low, a small manufacturer will obtain much more profit than the large manufacturer; when the carbon price exceeds a threshold, the large manufacturer will obtain more profit than the small manufacturer. Additionally, the Stackelberg game is more beneficial to small manufacturer compared with the Nash game.

### 5.2. Impacts of Consumer Environmental Awareness on Optimal Emission Reduction Rates, Retail Prices and Profits

**Figure 4:**
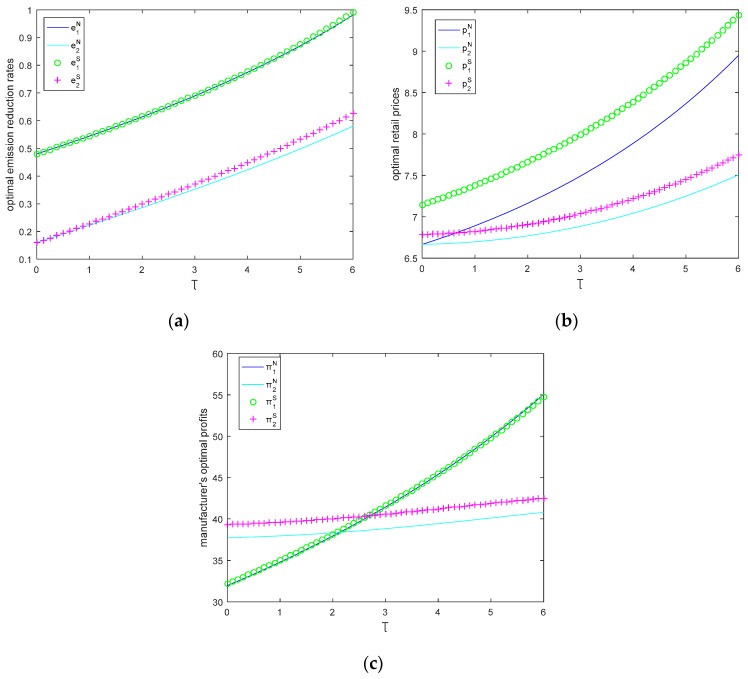
(**a**) Optimal emission reduction rates, (**b**) optimal retail prices, and (**c**) manufacturer’s profits as a function of *τ*.

In this section, let θ=0.5, $M_{1}=6$, $M_{2}=2$, $c_{0}=8$. We will study the impact of CEA on optimal retail price, emission reduction rate, and profit in Figure 4.

Figure 4 shows that as CEA increases, emission reduction rates, retail prices, and profits of the two manufacturers increase in both models. Due to CEA increases, the demand of the green product increases, and then manufacturers are more willing to provide more eco-friendly products to obtain more consumers.

We can also find that the carbon emission reduction rates in two models are almost the same, but the differences of each manufacturer’s price in two models is much larger, and the price with Stackelberg game model is much larger than that in Nash game model, and manufacturer 1’s profits in two models are almost the same, but manufacturer 2 may obtain more profit in Stackelberg game. Additionally, the manufacturer with small size may not mean that he will earn low profit, especially when CEA is much lower (τ<2.3 in Nash game model, τ<2.8 in Stackelberg game model), the smaller manufacturer will obtain much more profit than the larger manufacturer; when CEA is much bigger, the larger manufacturer will obtain more profit than the smaller manufacturer.

Therefore, CEA affects both manufacturers’ profits: CEA increases both manufacturers’ profits; when CEA is much lower, the small manufacturer has more advantage than the large manufacturer; otherwise, the large manufacturer has more advantage.

### 5.3. Impacts of Manufacturer 1’s Size on Optimal Emission Reduction Rates, Retail Prices and Profits

In this section, let τ=3, θ=0.5, $M_{2}=5$, $c_{0}=8$, we will study the impacts of manufacturer’s size on optimal retail prices, emission reduction rates, and profits. Due to the situation that the two manufacturers are symmetry, we only discuss the scenario when the size of the manufacturer 1 changes.

**Figure 5:**
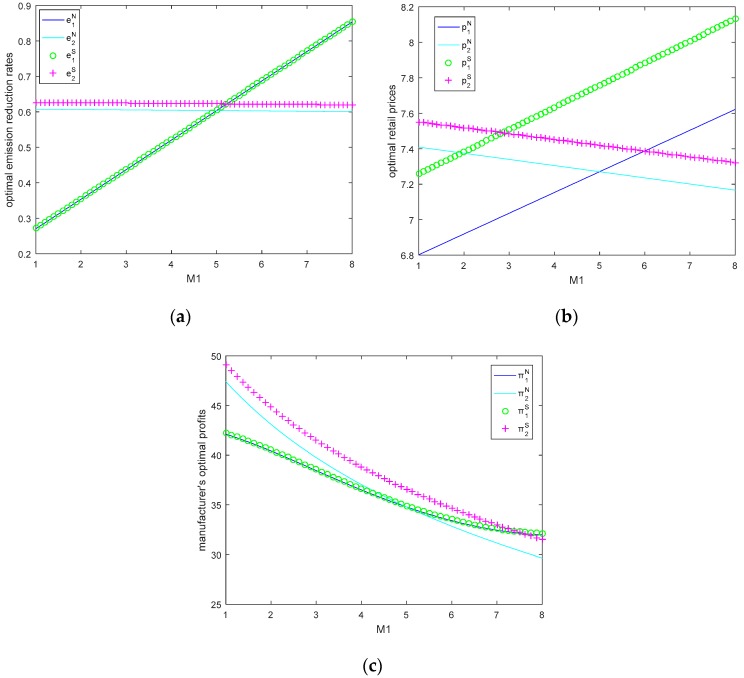
(**a**) Optimal emission reduction rates, (**b**) optimal retail prices, and (**c**) manufacturer’s profits as a function of M1.

From Figure 5a,b, we can see that manufacturer 1’s optimal emission reduction rates and retail prices rise significantly with M1 in both models. As manufacturer’s size increases, the carbon cap increases, but the manufacturer does not produce product with high emission, because the total emission increases with the manufacturer’s size.

Manufacturer 2’s optimal retail price reduces significantly, but optimal emission reduction rates are almost unchanged with manufacturer 1’s size. Two manufacturer’s prices in Stackelberg game model are larger than that in Nash game model. From Figure 5c, we can see that manufacturers’ optimal profits decrease with the size of the manufacturer 1 in both models. In the Nash game model, the bigger the size of the manufacturer, the larger manufacturer’s profit, but in the Stackelberg game model, bigger manufacturer size does not mean bigger profit.

Therefore, the manufacturer’s profit is affected by the manufacturer’s size and the decision sequence. When the two manufacturer have the same size, in Nash equilibrium model, the two manufacturers’ optimal retail prices, emission reduction rates, and profits are equal, but in Stackelberg game model, we can see that manufacturer 2’s retail price is lower than manufacturer 1, but manufacturer 2’s emission reduction rate and profit are higher than manufacturer 1, in other word, when two manufacturers have the same size, the manufacturer have late move advantage.

### 5.4. Impacts of the Sensitivity of Switchovers Toward Price on Optimal Emission Reduction Rates, Retail Prices and Profits

In this section, we set τ=3, $M_{1}=6$, $M_{2}=2$, $c_{0}=8$. We will study the impacts of the sensitivity of switchovers toward price on optimal emission reduction rate, retail price, and profit.

**Figure 6:**
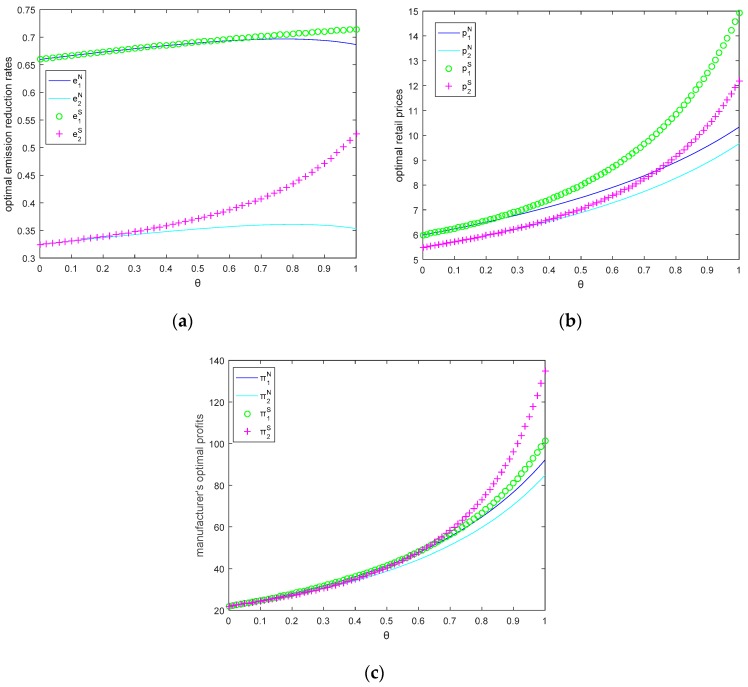
(**a**) Optimal emission reduction rates, (**b**) optimal retail prices and (**c**) manufacturer’s profits as a function of *θ*.

From Figure 6, we can see that the manufacturers’ optimal retail prices and optimal profits increase with the sensitivity of switchovers toward price in both models and manufacturer 1 with large size produces much greener product. As the sensitivity of switchovers toward price also represents that the sensitivity towards low emission (high green quality), and then manufacturers will produce product with high green quality.

In Nash game model, manufacturer 1’s profit is always bigger than that of the manufacturer 2 with θ, but in Stackelberg game model, first manufacturer 1’s profit is bigger than that of manufacturer 2, then manufacturer 1’s profit is smaller than that of manufacturer 2 with θ. In other words, the bigger size does not mean larger profit.

In sum, Stackelberg game leads manufacturers to produce more eco-friendly products than the Nash game and the leader with big size produces much greener products than the follower, and the Stackelberg game is much more beneficial to the follower than the leader.

## 6. Conclusions

This study examines the impacts of CCT-mechanism, CEA, and manufacturers’ competition on optimal carbon emission reduction rates, retail prices, and manufacturers’ profits. When each manufacturer’s carbon cap is determined by manufacturers’ sizes, and the demand depends on both the emission rates and retail prices, this paper gives the optimal emission rates and prices for the Nash and Stackelberg game models. By the sensitivity analysis, this paper compares the optimal solutions in two models and presents the change trends with some important parameters. Our study has four main managerial implications.

First, carbon price is critical to manufacturers’ decision-making when the CCT-mechanism is implemented in the market. There is a trade off between investing in greener product and purchasing carbon quota. When the carbon price is not high, the manufacturer tends to purchase the carbon quota; and when the carbon price is much higher, the manufacturer is more willing to increase the environmental quality of the product. From the perspective of environmental protection, higher carbon price is conducive to reduce carbon emissions, but high carbon price will put manufacturers at a disadvantage. Therefore, the government should set a proper carbon price to balance the environment and the economy.

Second, high consumer environmental awareness leads to low carbon emission and high profits in both models. Manufacturers and government could cooperate to invest more in advertising or other ways to improve CEA. It will not only help firms obtain more profits, but also reduce carbon emissions. Therefore, raising CEA is a primary measure to benefit both manufacturers and the environment.

Third, the decision sequence changes manufacturer’s decisions. The optimal emission reduction rates in Nash and Stackelberg game models are almost the same, but the prices in different models have big differences. Additionally, prices in Stackelberg game model are much larger than those in Nash game model. Manufacturer 1’s profits in two models are almost same, but manufacturer 2 may obtain more profit in Stackelberg game, in other words, the manufacturer has late move advantage in Stackelberg game.

Fourth, manufacturer’s size affects product’s emission reduction rate and manufacturer’s optimal profit. The larger manufacturer tends to produce much greener product. But large manufacturer does not mean that he could obtain much more money than the small manufacturer. The lower carbon price, the lower CEA or the higher sensitivity of switchovers toward price may make the large manufacturer earn less profit than the small manufacturer.

This paper can be extended in some directions. It is common to find multiple competitive manufacturers, hence multi-manufacturer production optimization and competition problems under Carbon Cap and Trade could be studied. Another extension of our work is to explore the effects of CCT-mechanism on supply chain. It would be interesting to find certain effective contracts to coordinate the supply chain and to investigate the impact of competition among manufacturers on contract efficiency.

## Figures and Tables

**Table 1:** Comparison of previous models with this study.

Authors	Carbon Emission Reduction Level	CEA	Carbon Cap and Trade Mechanism	Competition	Nash Equilibrium Game	Stackelberg Game
Liu et al. (2012)	√	√		√		√
Zhang et al. (2015)	√	√		√		√
Dong et al. (2016)	√		√			√
Xu et al. (2016)	√	√				√
Wang et al. (2016)	√	√	√			√
Hammami et al. (2017)	√	√	√			
Cheng et al. (2017)	√		√		√	√
Ji et al. (2017)	√	√	√			√
Zhu et al. (2017)	√	√		√		√
This paper	√	√	√	√	√	√

**Table 2:** Model parameters and Decision variables (in this paper, right subscript *i* = 1, 2).

**Parameters**	
$m_{i}$	the manufacturer *i* ($m_{i}>0$)
c	cost coefficient of emission reduction of the manufacturer (c>0)
τ	consumer environmental awareness (CEA)
θ	the sensitivity of switchovers toward price (θ∈[0,1])
$c_{0}$	unit price of carbon emission ($c_{0}>0$)
a	initial market potential (a≥0)
$M_{i}$	the size of the manufacturer *i* ($M_{i}>0$)
$s_{0}$	the government’s total carbon quota ($s_{0}>0$)
$\pi_{i}$	the expected manufacturer *i*’s profit
**Decision variables**	
$e_{i}$	emission reduction rate of product *i* ($e_{i}\in \left[0,1\right]$)
$p_{i}$	retail price per unit of manufacturer *i* ($p_{i}>0$)

## Appendix A

**Proof of Theorem 1.** From Equation (4), we get

$$\frac{\partial \pi_{2}}{\partial p_{2}}=a-2p_{2}+\theta p_{1}-\tau \theta e_{1}+\tau e_{2};$$

$$\frac{\partial \pi_{2}}{\partial e_{2}}=p_{2}\tau -2ce_{2}+c_{0}M_{2}.$$

We can conclude that

$$\frac{\partial^{2}\pi_{2}}{\partial p_{2}^{2}}=-2<0;$$

$$\frac{\partial^{2}\pi_{2}}{\partial e_{2}^{2}}=-2c;$$

$$\frac{\partial^{2}\pi_{2}}{\partial p_{2}\partial e_{2}}=\frac{\partial^{2}\pi_{2}}{\partial e_{2}\partial p_{2}}=\tau .$$

Then we get that

$$|\begin{array}{cc} \frac{\partial^{2}\pi_{2}}{\partial p_{2}^{2}} & \frac{\partial^{2}\pi_{2}}{\partial p_{2}\partial e_{2}}\\ \frac{\partial^{2}\pi_{2}}{\partial e_{2}\partial p_{2}} & \frac{\partial^{2}\pi_{2}}{\partial e_{2}^{2}} \end{array}|=4c-\tau^{2}>0.$$

Thus, when $c>\frac{\tau^{2}}{4}$, $\pi_{2}$ is a joint concave function of $p_{2}$ and $e_{2}$. Similarly, from Equation (3), we get

$$\frac{\partial \pi_{1}}{\partial p_{1}}=a-2p_{1}+\theta p_{2}-\tau \theta e_{2}+\tau e_{1};$$

$$\frac{\partial \pi_{1}}{\partial e_{1}}=p_{1}\tau -2ce_{1}+c_{0}M_{1}.$$

We can conclude that:

$$\frac{\partial^{2}\pi_{1}}{\partial p_{1}^{2}}=-2<0;$$

$$\frac{\partial^{2}\pi_{1}}{\partial e_{1}^{2}}=-2c;$$

$$\frac{\partial^{2}\pi_{1}}{\partial p_{1}\partial e_{1}}=\frac{\partial^{2}\pi_{1}}{\partial e_{1}\partial p_{1}}=\tau .$$

Then we get

$$|\begin{array}{cc} \frac{\partial^{2}\pi_{1}}{\partial p_{1}^{2}} & \frac{\partial^{2}\pi_{1}}{\partial p_{1}\partial e_{1}}\\ \frac{\partial^{2}\pi_{1}}{\partial e_{1}\partial p_{1}} & \frac{\partial^{2}\pi_{1}}{\partial e_{1}^{2}} \end{array}|=4c-\tau^{2}>0.$$

Thus, when $c>\frac{\tau^{2}}{4}$, $\pi_{1}$ is a joint concave function of $p_{1}$ and $e_{1}$. We can draw a conclusion: two manufacturers’ profit on the retail price is a concave function, there are optimal retail price $p_{1}^{N*}$ and $p_{2}^{N*}$ to make two manufacturers earns the largest income respectively, and the optimal price $p_{1}^{N*}$ and $p_{2}^{N*}$ are the global optimum. Let $\frac{\partial \pi_{1}}{\partial p_{1}}=0$, $\frac{\partial \pi_{2}}{\partial p_{2}}=0$, thus we obtain Theorem 1. □

**Proof of Proposition 1.** According to Equations (5) and (6), and we have already known: τ≥0; θ∈[0,1], therefore 1 ≤ $2-\theta^{2}$ ≤ 2; 3 ≤ $4-\theta^{2}$ ≤ 4. Therefore, we have

$$\frac{\partial p_{1}^{N*}}{\partial e_{1}}=\frac{\partial p_{2}^{N*}}{\partial e_{2}}=\frac{\tau \left(2-\theta^{2}\right)}{4-\theta^{2}}\geq 0;$$

$$\frac{\partial p_{1}^{N*}}{\partial e_{2}}=\frac{\partial p_{2}^{N*}}{\partial e_{1}}=\frac{-\tau \theta }{4-\theta^{2}}\leq 0.$$

□

**Proof of Proposition 2.** We have known $c>\frac{\tau^{2}}{4}$, according to Equations (5) and (6), let

$$\frac{\partial p_{1}^{N*}}{\partial \tau }\geq 0.$$

We can get

$$\frac{\partial p_{1}^{N*}}{\partial \tau }=\frac{2e_{1}-e_{1}\theta^{2}-e_{2}\theta }{4-\theta^{2}}\geq 0.$$

We have known $3\leq 4-\theta^{2}\leq 4$, therefore if

$$\frac{\partial p_{1}^{N*}}{\partial \tau }=\frac{2e_{1}-e_{1}\theta^{2}-e_{2}\theta }{4-\theta^{2}}\geq 0.$$

It is equivalent to

$$2e_{1}-e_{1}\theta^{2}-e_{2}\theta \geq 0.$$

We can get

$$\frac{e_{1}}{e_{2}}\geq \frac{\theta }{2-\theta^{2}}.$$

Thus we obtain Proposition 2 (i). Similarly we can obtain Proposition 2 (ii) easily. □

**Proof of Proposition 3.** We substitute the Equations (5) and (6) into (3) and (4). We can get the optimal manufacturer’ profit and retailer’s profit.

$$\pi_{1}^{N*}=\frac{{\left(2a+a\theta +2e_{1}\tau -e_{1}\tau \theta^{2}-e_{2}\tau \theta \right)}^{2}}{{\left(\theta^{2}-4\right)}^{2}}-ce_{1}^{2}+\frac{M_{1}c_{0}\left(s_{0}-M_{2}-M_{1}+M_{1}e_{1}+M_{2}e_{1}\right)}{M_{1}+M_{2}};$$

$$\pi_{2}^{N*}=\frac{{\left(2a+a\theta +2e_{2}\tau -e_{2}\tau \theta^{2}-e_{1}\tau \theta \right)}^{2}}{{\left(\theta^{2}-4\right)}^{2}}-ce_{2}^{2}+\frac{M_{2}c_{0}\left(s_{0}-M_{2}-M_{1}+M_{1}e_{2}+M_{2}e_{2}\right)}{M_{1}+M_{2}}.$$

Let $\frac{\partial \pi_{1}^{N*}}{\partial c_{0}}\geq 0$, we get

$$\frac{\partial \pi_{1}^{N*}}{\partial c_{0}}=\frac{M_{1}}{M_{1}+M_{2}}s_{0}+M_{1}\left(e_{1}-1\right)\geq 0.$$

Therefore $e_{1}\geq 1-\frac{s_{0}}{M_{1}+M_{2}}$. Let $\frac{\partial \pi_{1}^{N*}}{\partial c_{0}}<0$, we get

$$\frac{\partial \pi_{1}^{N*}}{\partial c_{0}}=\frac{M_{1}}{M_{1}+M_{2}}s_{0}+M_{1}\left(e_{1}-1\right)<0.$$

Therefore $e_{1}<1-\frac{s_{0}}{M_{1}+M_{2}}$. We have known $e_{i}\in \left[0,1\right]$, Thus we obtain Proposition 3 (i). Similarly we can obtain Proposition 3 (ii) easily. □

**Proof of Proposition 4.** Let $\frac{\partial \pi_{1}^{N*}}{\partial c_{0}}\geq 0$, we can get

$$\frac{\partial \pi_{1}^{N*}}{\partial c_{0}}=\frac{M_{1}s_{0}}{M_{1}+M_{2}}-M_{1}+M_{1}e_{1}\geq 0;$$

$$0\leq e_{1}\leq 1,\;e_{1}-1\leq 0.$$

Therefore if $\frac{\partial \pi_{1}^{N*}}{\partial c_{0}}=\frac{M_{1}s_{0}}{M_{1}+M_{2}}-M_{1}+M_{1}e_{1}\geq 0$, we get

$$\frac{M_{1}s_{0}}{M_{1}+M_{2}}\geq M_{1}-M_{1}e_{1}.$$

It is equivalent to

$$\frac{s_{0}}{M_{1}+M_{2}}\geq 1-e_{1}.$$

Therefore

$$1-\frac{s_{0}}{M_{1}+M_{2}}\leq e_{1}\leq 1.$$

Let $\frac{\partial \pi_{1}^{N*}}{\partial c_{0}}<0$, we can get

$$\frac{\partial \pi_{1}^{N*}}{\partial c_{0}}=\frac{M_{1}s_{0}}{M_{1}+M_{2}}-M_{1}+M_{1}e_{1}<0;$$

$$0\leq e_{1}\leq 1,\;e_{1}-1\leq 0.$$

Therefore if $\frac{\partial \pi_{1}^{N*}}{\partial c_{0}}=\frac{M_{1}s_{0}}{M_{1}+M_{2}}-M_{1}+M_{1}e_{1}<0$, we get

$$\frac{M_{1}s_{0}}{M_{1}+M_{2}}<M_{1}-M_{1}e_{1}.$$

It is equivalent to

$$\frac{s_{0}}{M_{1}+M_{2}}<1-e_{1}.$$

Therefore

$$0\leq e_{1}<1-\frac{s_{0}}{M_{1}+M_{2}}.$$

We have known $0\leq 1-\frac{s_{0}}{M_{1}+M_{2}}<1$, therefore $0<s_{0}\leq M_{1}+M_{2}$. Thus we obtain Proposition 4 (i). Similarly we can obtain Proposition 4 (ii). □

**Proof of Proposition 5.** According to the optimal manufacturer’ profit and retailer’s profit, we get

$$\frac{\partial \pi_{1}^{N*}}{\partial M_{2}}=-\frac{c_{0}s_{0}}{{\left(M_{1}+M_{2}\right)}^{2}};$$

We have known $c_{0}>0$, $s_{0}>0$, $M_{i}>0$, therefore $\frac{\partial \pi_{1}^{N*}}{\partial M_{2}}<0$. Thus we obtain Proposition 5 (i). Similarly we can obtain Proposition 5 (ii) easily. □

**Proof of Theorem 2.** According to the optimal manufacturer’ profit and retailer’s profit and proof of Theorem 1, two manufacturers’ profit on the emission reduction rate is a concave function, there are optimal emission reduction rate $e_{1}^{N*}$ and $e_{2}^{N*}$ to make two manufacturers earns the largest income respectively, and the optimal emission reduction rate $e_{1}^{N*}$ and $e_{2}^{N*}$ are the global optimum. Let $\frac{\partial \pi_{1}^{N*}}{\partial e_{1}}=0$, $\frac{\partial \pi_{2}^{N*}}{\partial e_{2}}=0$, thus we obtain Theorem 2. □

**Proof of Theorem 3.** From Equation (4), we get

$$\frac{\partial \pi_{2}}{\partial p_{2}}=a-2p_{2}+\theta p_{1}-\tau \theta e_{1}+\tau e_{2};$$

$$\frac{\partial \pi_{2}}{\partial e_{2}}=p_{2}\tau -2ce_{2}+c_{0}M_{2}.$$

We can conclude that:

$$\frac{\partial^{2}\pi_{2}}{\partial p_{2}^{2}}=-2<0;$$

$$\frac{\partial^{2}\pi_{2}}{\partial e_{2}^{2}}=-2c;$$

$$\frac{\partial^{2}\pi_{2}}{\partial p_{2}\partial e_{2}}=\frac{\partial^{2}\pi_{2}}{\partial e_{2}\partial p_{2}}=\tau .$$

Then we get

$$|\begin{array}{cc} \frac{\partial^{2}\pi_{2}}{\partial p_{2}^{2}} & \frac{\partial^{2}\pi_{2}}{\partial p_{2}\partial e_{2}}\\ \frac{\partial^{2}\pi_{2}}{\partial e_{2}\partial p_{2}} & \frac{\partial^{2}\pi_{2}}{\partial e_{2}^{2}} \end{array}|=4c-\tau^{2}>0.$$

Thus, when $c>\frac{\tau^{2}}{4}$, $\pi_{2}$ is a joint concave function of $p_{2}$ and $e_{2}$. We substitute $e_{2}^{S*}$ and $p_{2}^{S*}$ into Equation (3), we can conclude that:

$$\frac{\partial^{2}\pi_{1}}{\partial p_{1}^{2}}=\frac{-2\left(\tau^{2}\theta^{2}-\tau^{2}-2c\theta^{2}+4c\right)}{4c-\tau^{2}}<0;$$

$$\frac{\partial^{2}\pi_{1}}{\partial e_{1}^{2}}=-2c;$$

$$\frac{\partial^{2}\pi_{1}}{\partial p_{1}\partial e_{1}}=\frac{\partial^{2}\pi_{1}}{\partial e_{1}\partial p_{1}}=\frac{\tau \left(\tau^{2}\theta^{2}-\tau^{2}-2c\theta^{2}+4c\right)}{4c-\tau^{2}}.$$

Then we get

$$|\begin{array}{cc} \frac{\partial^{2}\pi_{1}}{\partial p_{1}^{2}} & \frac{\partial^{2}\pi_{1}}{\partial p_{1}\partial e_{1}}\\ \frac{\partial^{2}\pi_{1}}{\partial e_{1}\partial p_{1}} & \frac{\partial^{2}\pi_{1}}{\partial e_{1}^{2}} \end{array}|=\frac{4c\left(\tau^{2}\theta^{2}-\tau^{2}-2c\theta^{2}+4c\right)}{4c-\tau^{2}}-\frac{\tau^{2}{\left(\tau^{2}\theta^{2}-\tau^{2}-2c\theta^{2}+4c\right)}^{2}}{{\left(4c-\tau^{2}\right)}^{2}}>0.$$

Thus, when $c>\frac{\tau^{2}}{4}$, we get $\pi_{2}$ is a joint concave function of $p_{2}$ and $e_{2}$. It is equivalent to

$$\frac{4c\left(\tau^{2}\theta^{2}-\tau^{2}-2c\theta^{2}+4c\right)}{4c-\tau^{2}}>\frac{\tau^{2}{\left(\tau^{2}\theta^{2}-\tau^{2}-2c\theta^{2}+4c\right)}^{2}}{{\left(4c-\tau^{2}\right)}^{2}}.$$

$\pi_{1}$ is a joint concave function of $p_{1}$ and $e_{1}$. Let $\frac{\partial \pi_{2}}{\partial p_{2}}=\frac{\partial \pi_{2}}{\partial e_{2}}=0$, we can get Theorem 3, similarly proof of Theorem 1 and 2, the optimal retail price and emission reduction rate of manufacturer 2 are the global optimum. □

**Proof of Proposition 6.** According to Equations (9) and (10), we get

$$\frac{\partial e_{2}^{S*}}{\partial c_{0}}=\frac{2M_{2}}{4c-\tau^{2}}>0;$$

$$\frac{\partial p_{2}^{S*}}{\partial c_{0}}=\frac{M_{2}\tau }{4c-\tau^{2}}>0.$$

Thus we obtain Proposition 6. □

**Proof of Proposition 7.** According to Equations (9) and (10), we get

$$\frac{\partial e_{2}^{S*}}{\partial M_{2}}=\frac{2c_{0}}{4c-\tau^{2}}\geq 0;$$

$$\frac{\partial p_{2}^{S*}}{\partial M_{2}}=\frac{c_{0}\tau }{4c-\tau^{2}}\geq 0.$$

Thus we obtain Proposition 7. □

**Proof of Proposition 8.** According to Equations (9) and (10), we get

$$\frac{\partial e_{2}^{S*}}{\partial e_{1}}=\frac{-\tau^{2}\theta }{4c-\tau^{2}}\leq 0;$$

$$\frac{\partial p_{2}^{S*}}{\partial e_{1}}=\frac{-2c\tau \theta }{4c-\tau^{2}}\leq 0;$$

$$\frac{\partial e_{2}^{S*}}{\partial p_{1}}=\frac{\tau \theta }{4c-\tau^{2}}\geq 0;$$

$$\frac{\partial p_{2}^{S*}}{\partial p_{1}}=\frac{2c\theta }{4c-\tau^{2}}\geq 0.$$

Thus we obtain Proposition 8. □

**Proof of Proposition 9.** According to Equations (9) and (10), we get

$$\frac{\partial e_{2}^{s*}}{\partial \theta }=\frac{\tau \left(p_{1}-e_{1}\tau \right)}{4c-\tau^{2}}.$$

We have known τ≥0, $4c-\tau^{2}>0$, therefore if $\frac{\partial e_{2}^{s*}}{\partial \theta }=\frac{\tau \left(p_{1}-e_{1}\tau \right)}{4c-\tau^{2}}\geq 0$, is equivalent to $\tau \leq \frac{p_{1}}{e_{1}}$. $4c-\tau^{2}>0$ is equivalent to $\tau <2\sqrt{c}$. if $\frac{p_{1}}{e_{1}}<2\sqrt{c}$, we can get Proposition 9 (i). Similarly if $\frac{p_{1}}{e_{1}}\geq 2\sqrt{c}$, and $\tau <2\sqrt{c}$, we can get Proposition 9 (ii) easily. Thus we obtain Proposition 9. □

**Proof of Theorem 4.** According to Equations (1)–(3) and Theorem 3, we substitute the Equations (9) and (10) into the Equation (3), we get

$$\pi_{1}^{\prime }=p_{1}\left(a-p_{1}+\theta p_{2}^{S*}+\tau e_{1}-\tau \theta e_{2}^{S*}\right)-ce_{1}^{2}+\left(\frac{M_{1}}{M_{1}+M_{2}}s_{0}-M_{1}\left(1-e_{1}\right)\right)c_{0}\;$$

When $\frac{4c\left(\tau^{2}\theta^{2}-\tau^{2}-2c\theta^{2}+4c\right)}{4c-\tau^{2}}>\frac{\tau^{2}{\left(\tau^{2}\theta^{2}-\tau^{2}-2c\theta^{2}+4c\right)}^{2}}{{\left(4c-\tau^{2}\right)}^{2}}$, let $\frac{\partial \pi_{1}^{\prime }}{\partial p_{1}}=\frac{\partial \pi_{1}^{\prime }}{\partial e_{1}}=0$, we can get Theorem 4, similarly Theorem 1–3, the optimal retail price and emission reduction rate of manufacturer 1 are the global optimum. □
